# One-Pot Microwave-Assisted Synthesis of Graphene/Layered Double Hydroxide (LDH) Nanohybrids

**DOI:** 10.1007/s40820-015-0047-3

**Published:** 2015-06-30

**Authors:** Sunil P. Lonkar, Jean-Marie Raquez, Philippe Dubois

**Affiliations:** 1 Laboratory of Polymeric and Composite Materials, Center of Innovation and Research in Materials and Polymers, University of Mons, Place du Parc 23, 7000 Mons, Belgium; 2 Department of Chemical Engineering, The Petroleum Institute, P.O Box 2533, Abu Dhabi, United Arab Emirates

**Keywords:** Graphene, LDHs, Nanostructures, Microwave, Composite materials

## Abstract

A facile and rapid method to synthesize graphene/layered double hydroxide (LDH) nanohybrids by a microwave technique is demonstrated. The synthesis procedure involves hydrothermal crystallization of Zn–Al LDH at the same time in situ reduction of graphene oxide (GO) to graphene. The microstructure, composition, and morphology of the resulting graphene/LDH nanohybrids were characterized. The results confirmed the formation of nanohybrids and the reduction of graphene oxide. The growth mechanism of LDH and in situ reduction of GO were discussed. The LDH sheet growth was found to prevent the scrolling of graphene layers in resulting hybrids. The electrochemical properties exhibit superior performance for graphene/Zn–Al LDH hybrids over pristine graphene. The present approach may open a strategy in hybridizing graphene with multimetallic nano-oxides and hydroxides using microwave method.

## Introduction

The combination of multidimensional nanomaterials leads to the formation of hierarchical composites that can take full advantages of each kind of components, which is an effective way for the preparation of multifunctional materials with exceptional properties. Recently, nanocarbon (e.g., carbon nanotubes and graphene sheets) has emerged as the most powerful material and was used in multifunctional hybrids for various applications [1–3]. Among nanocarbon materials, graphene has attracted a great deal of interests because of its single-atom thick, unique, and extensively conjugated structure, which exhibits intriguing properties like excellent electrical, thermal conductivities, and high stiffness [4, 5]. However, their inter-structural affinity leads to an irreversible agglomeration during their synthesis that amends intrinsic properties of graphene sheets, therefore confining their applicability [6]. The retention of layered structure is vital for graphene nanosheets because most of their unique properties are primarily associated with individual sheets. Hence, to overcome this issue, hybridizing graphene with substrates like metals, metal oxides, and polymers are being practiced for various applications [7, 8].

Recently, decorating layered metal hydroxides like layered double hydroxides (LDHs) with different metal compositions have been studied in order to diminish the restacking interactions and to limit the aggregation in graphene nanosheets suitable for electrochemical applications [9, 10]. LDHs are brucite-like solids that are mainly constituted by two metals typically having 2 + (MII) and 3 + (MIII) or 4 + (MIV) oxidation states, octahedrally surrounded by oxo bridges and hydroxyl groups. The structure is organized as nanometer-thick layers that bear an excess of positive charge equivalent to the number of trivalent or twice the tetravalent metal compensated by anions that are located in the intergallary spaces [11]. Because of their high surface area, variable metal compositions, and anion exchange property, LDH materials have been widely employed in a large set of applications [12, 13]. Especially, the LDHs composed of transition metals were explored as promising electrode materials in electrochemical field because of their relatively low cost, high redox activity, and environmentally friendly nature [14, 15]. Hence, hybridizing LDH nanosheets with large surface area in conjunction with thermo-electro conductive graphene can endow hybrid nanocomposites new multifunctional properties [16, 17]. However, most of the reported synthesis methods use hydrothermal process, which involves the use of high temperature aging over a long period of time, i.e., a process consuming high energy and time. Therefore, an alternative approach in synthesizing such multifunctional hybrids based on a rapid and facile method is highly desirable.

Microwave irradiation is often applied for the rapid synthesis of inorganic solids and organic synthetic reactions [18, 19]. The use of a microwave technique in LDHs synthesis over conventional hydrothermal process is gaining importance, and has shown to be a reliable technique to achieve highly crystalline layered structures. The microwave heating showed an enhancement of the crystallization rate of solids by improving the dissolution/recrystallization mechanism (Ostwald ripening), without the segregation of side phases [20]. However, few recent reports have shed light on the use of microwave technique in preparation of graphene [21, 22]. Therefore, to the best of our knowledge, no studies related to the preparation of graphene/LDH hybrids using the microwave synthesis method have been reported.

In this work, we present a one-step synthesis method to synthesize graphene/Zn–Al LDH hybrids through microwave-assisted growth of 2D LDHs with simultaneous in situ reduction of graphene oxide (GO) to graphene under hydrothermal conditions. In this facile and rapid synthetic procedure, the exfoliated GO was reduced to graphene using in situ hydrolyzed urea (ammonia). Simultaneously, the Zn–Al LDH platelets were formed in situ and hybridized with graphene. The resulting hybrids were characterized using various physicochemical characterization techniques. Furthermore, we also demonstrated the use of graphene/Zn–Al LDHs for electrochemical applications by studying their cyclic voltammetry (CV) and galvanostatic charge/discharge measurements.

## Experimental

### Synthesis of Reduced Graphene Oxide (RGO)/Zn–Al LDH Hybrids

All the chemicals were of analytical reagent grade and without any further purification. GO was prepared from expanded graphite by a modified Hummers method according to our previous report [23]. Exfoliation of GO was achieved by ultrasonication of the dispersion in an ultrasonic bath (Brandsonic 2210 E-MTH).

**Fig. 1:**
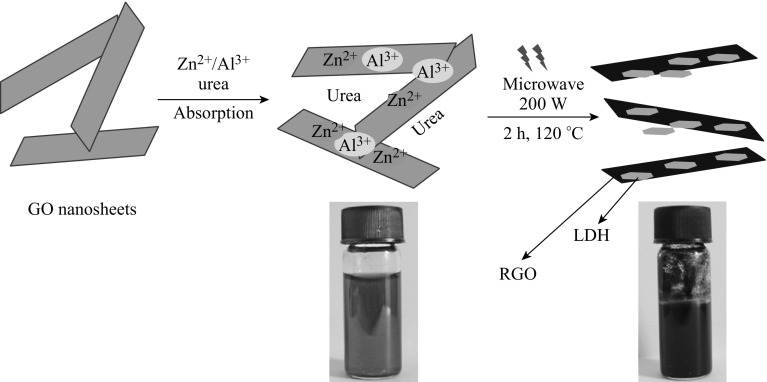
Schematic representation of graphene/LDH nanohybrids synthesis using microwave technique

The RGO/LDH nanohybrids were synthesized using urea-hydrolysis reaction under hydrothermal conditions using microwave technique (Fig. 1). In detail, 50 mL aqueous GO dispersion (1 mg mL^−1^) was sonicated for 5 min and placed in a microwave flask, and subsequently zinc nitrate (10 mmol) and aluminum nitrate (0.33 mmol) salt mixture (molar ratio of Zn/Al = 3) was added. The mixture was magnetically stirred for 30 min in order to form a colloidal dispersion. Subsequently, an excess urea (67 mmol, five times of Zn/Al salt concentration) was added to adjust the pH and to reduce GO through urea hydrolysis under microwave. Lastly, the flask containing Zn salt/GO dispersion was placed into microwave system (CEM Discover microwave system, USA) with cooling water circulating condenser. The system was heated gradually to 150 °C under microwave irradiation at a power of 200 W for 2 h. After cooling, the resulting black precipitate was repeatedly washed with CO_2_-free water via centrifugation before freeze drying. Finally, the resulting product was vacuum dried at 80 °C to remove any remaining traces of water. For the sake of comparison, pure RGO and Zn–Al LDH were also synthesized by under similar microwave-assisted technique using GO dispersion and metal salts, respectively.

### Characterization Techniques

The surface morphology, structure, and composition of graphene and graphene–LDH nanohybrids were characterized by transmission electron microscopy (TEM, Philips CM200 with a tungsten filament ken with CCD Gatan digital camera) and X-ray diffraction (XRD, Siemens D5000). The Raman spectra were obtained by a Renishaw Raman system Model 3000 spectrometer equipped with an integral microscope (Olympus BH2-UMA). Radiation from a He–Ne laser (633 nm) was used as the excitation source. The FT-IR was recorded on a Bruker Infrared Spectrometer using 32 scans and a 4 cm^−1^ resolution. Thermogravimetric analysis (TGA) of the resulting hybrids was studied by a TA instruments Q500 TGA at a heating rate of 10°C min^−1^ under inert atmosphere. X-ray photoelectron spectroscopy (XPS) was used to control the elemental composition of the samples. All reported spectra were recorded at a 90° take-off angle relative to the substrate with a VG ESCALAB 220iXL spectrometer using the monochromatized Al Kα radiation (1486.6 eV). The electrochemical properties of the nanohybrids were measured in an aqueous system (electrolyte: 30 % KOH). CV curves were measured with an electrochemistry workstation (Princeton PARSTAT2273). Galvanostatic charge/discharge measurement was conducted with a charge–discharge tester (PCBT-100-32D, Wuhan Lixing Testing Equipment Co., Ltd. China). The galvanostatic charge–discharge tests were performed on a BTS-5 V/10 mA battery-testing instrument (Neware, China) at room temperature. A two-electrode cell was assembled for cyclic testing of cell. During the cycling process, the cells were charged at 1C for 60 min and discharged at 1C down to 1.4 V cut-off voltages.

## Results and Discussion

### Synthesis

Figure 1 shows the schematic representation of RGO/LDH nanohybrids synthesis using microwave technique. The colloidal dispersion containing mixture of GO and Zn–Al metal salts was magnetically stirred to enable their adsorption within expanded interlayer spacing of GO sheets. This eventually allows metal cations to interact with oxygenated moieties from GO through electrostatic attraction. Urea was then added to the above mixture, which yielded ammonia upon hydrolysis as shown in the following reaction [24]: 

When evolved, ammonia increased the pH, resulting in the controlled precipitation of metal ions. Therefore, the use of microwave activation is expected to energize the metal cations, and through their inherent ionic conduction to achieve a uniform bulk during heating the materials. Moreover, the excess ammonia present in the system was also used as a reducing agent for GO reduction at higher temperature under microwave irradiation. During microwave heating, well-crystalized LDH nanoplatelets were thereby synthesized and closely interacted with the RGO layers. These nanoplatelets also prohibit the stacking of graphene sheets by van der Waals force as evidenced hereafter.

### Structure and Morphology

**Fig. 2:**
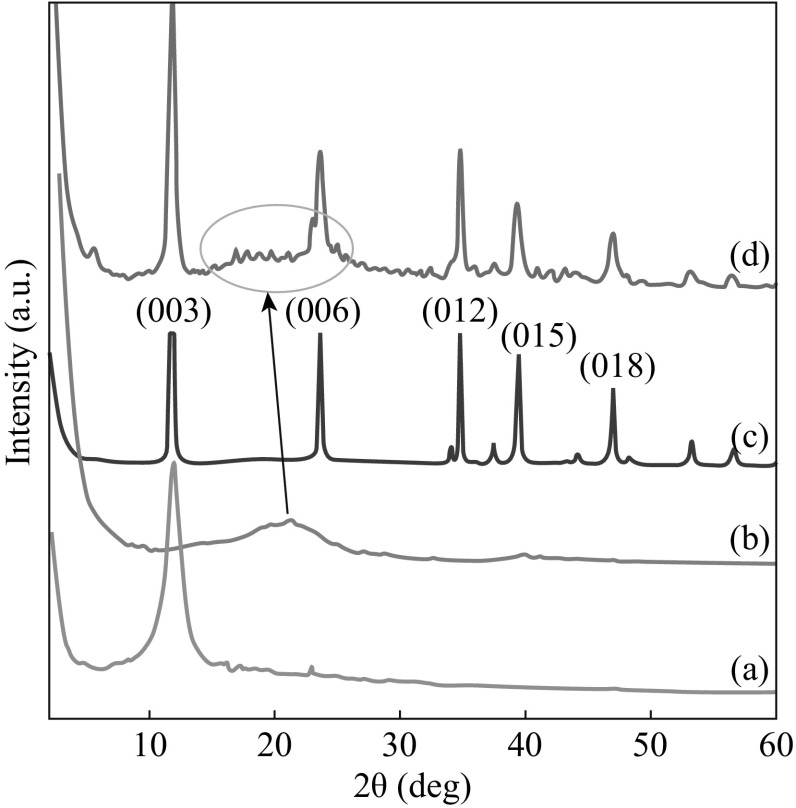
XRD patterns of* a* GO,* b* RGO,* c* Zn–Al LDH, and* d* RGO/Zn–Al LDH nanohybrids

Figure 2 shows the XRD patterns of GO, RGO, Zn–Al LDH, and the RGO/LDH nanohybrids. It can be seen that the diffraction peak of exfoliated GO at 11.7° (001) features a basal spacing of 0.75 nm, showing the complete oxidation of graphite to the GO due to the introduction of oxygen-containing functional groups on the graphene sheets (Fig. 2a). After in situ reduction of GO to RGO under microwave, most of the oxygen-containing moieties were eliminated. The XRD pattern of the RGO (Fig. 2b) shows the disappearance of the peak located at 11.7°, while the peak broadening at 2*θ* = 21° (002) was also observed, which reveals the reduction of GO to well-exfoliated RGO under microwave irradiation [25]. The interlayer spacing of the RGO changes from 0.75 nm for GO to 0.42 nm, which is still a bit larger than that of natural graphite (0.34 nm). This can be interpreted by the *π*–*π* stacking interactions between the graphene sheets, leading to the formation of the agglomerates. However, the residual oxygen functionalities on the RGO surface induce electrostatic repulsion, stabilizing the graphene sheets.

**Fig. 3:**
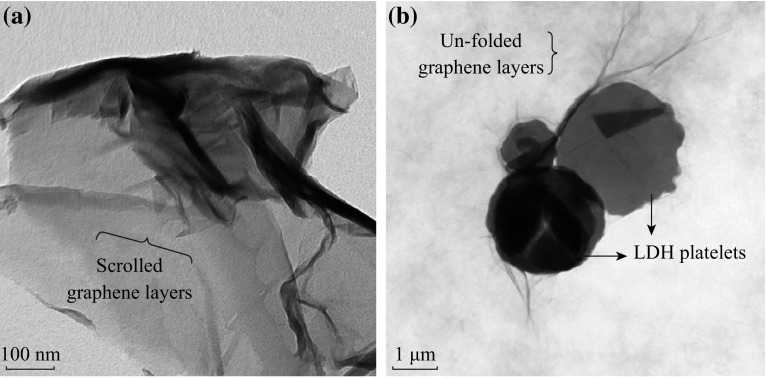
TEM micrographs of **a** RGO, and **b** RGO/Zn–Al RGO nanohybrids

These residual oxygenated functional groups most likely involve the intercalation and adsorption of metal ions onto the GNS. The XRD patterns of the as-prepared pure Zn–Al LDH and RGO/LDH nanohybrid materials (Fig. 2c) show typical peaks of hydrotalcite structure [26], while no characteristic peak of GO was observed. For RGO/LDH (Fig. 2d), the peak corresponding to layered graphene (002) was significantly broadened and shifted toward a lower *θ* value. The Zn–Al LDH crystals deposited on graphene can prevent them from stacking into multilayers, leading to the much lower crystalline extent of graphene, which results in the (002) peak broadening and shift toward lower angle. Moreover, the diffraction peak intensity of RGO/LDH nanohybrids nearly remains the same as of pure Zn–Al LDH. This means that under microwave irradiation, the crystallinity is improved with respect to the conventional hydrothermal method. This result suggests that the restacking of the graphene sheets is effectively prevented from a complete exfoliation state of graphene in the RGO/LDH nanohybrids, which is in well agreement with the morphological findings observed by TEM (Fig. 3).

Figure 3 shows the TEM images of graphene and graphene/LDH nanohybrids. The wavy and scrolled multilayer sheets were seen in case of RGO (Fig. 3a). However, the morphological features of RGO/LDH nanohybrids (Fig. 3b) show graphene sheets intimately interacting with the round-shaped LDH platelets, which somehow resembles silk blanket. Therefore, the microwave-assisted hybridization of graphene with LDHs can control the morphology of graphene sheets, which are less scrolled and display high surface area.

**Fig. 4:**
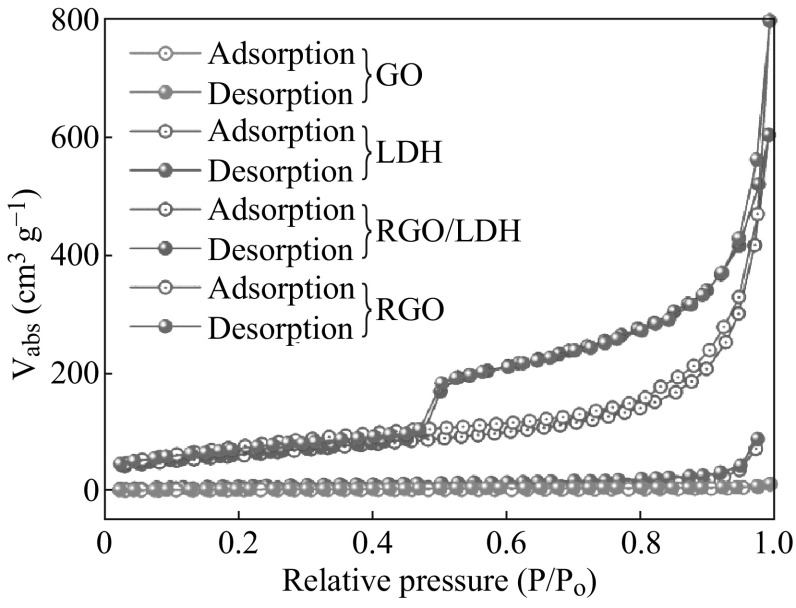
BET N_2_ adsorption–desorption loops for GO, LDH, RGO/LDH, and RGO

In order to confirm that the formation of graphene/Zn–Al LDH prevents the agglomeration of graphene nanosheets, nitrogen adsorption and desorption were carried out to obtain the BET-specific surface area of the samples. It was observed that the hybridization of graphene with LDH and in situ reduction of GO to RGO significantly increase the surface area. The corresponding specific surface area of graphene/Zn–Al LDH, Zn–Al LDH, and GO is about 223.4, 46.3, and 7.88 m^2^ g^−1^, respectively (Fig. 4).

**Fig. 5:**
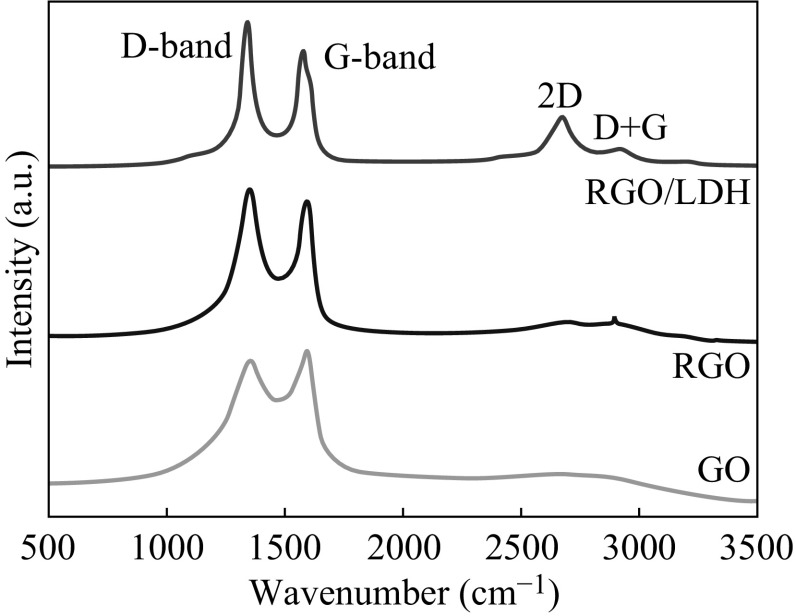
Raman spectra for* a* GO,* b* RGO, and* c* RGO/Zn–Al LDH

The GO reduction and simultaneous LDHs growth under microwave irradiation can be further confirmed by XPS spectroscopy as shown in Fig. 5. The XPS wide scan spectrum clearly indicates that the GO was successfully reduced to RGO under microwave treatment as the intensity of C 1*s* (284 eV) peak is significantly increased while O 1*s* (533) peak intensity has been gone down. For, RGO/Zn–Al LDH hybrids, the C 1 *s* and O 1 *s* peak intensities are matching to the RGO confirming the successful GO reduction to RGO. Furthermore, the spectrum also exhibits the peaks of Zn 2*p* and Al 2*p*, suggesting the formation of the RGO/Zn–Al LDH composite. The high-resolution C 1*s* spectrum of GO shows three types of carbon bonds (Fig. 5a). The peak of the non-oxygenated ring C (C–C) located at 284.4 eV is assigned to the bonds between the *sp* ^2^ hybridized carbon atoms, and the two peaks at 286.5 and 288.0 eV are attributed to the epoxy and alkoxy carbon (C–O) and the carboxylate carbon (O–C=O), indicating a considerable degree of oxidation [27]. The C 1*s* XPS spectrum of RGO/LDH (Fig. 5b) also exhibits similar types of carbon bondings. However, compared to that of GO, the absorbance band intensities of the epoxy and alkoxy carbon (C–O) and the carboxylate carbon (O–C=O) of the RGO/LDH composite decrease significantly due to the reduction of GO to graphene. By integrating the area of these peaks, the percentage of oxygen-containing ring C in the GO was calculated to be 51 %, whereas that of the RGO/LDH composite is 20 %. These results indicate that most of the oxygen-containing functional groups in the RGO/LDH hybrids are removed, thus confirming the successful reduction of GO to graphene. From XPS studies, the final ratio between RGO and LDH was obtained to be 84:16 wt%.

**Fig. 6:**
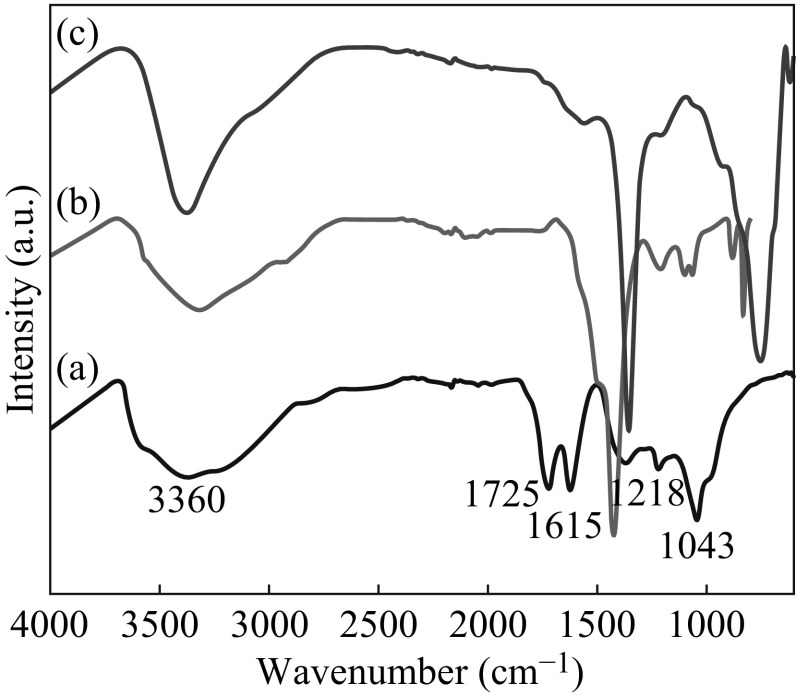
FT-IR spectra of* a* GO,* b* RGO/Zn–Al LDH, and* c* Zn–Al LDH

Further microstructural characteristics of the graphitic structure in GO, RGO, and RGO/LDH hybrids were revealed by Raman spectroscopy. As shown in Fig. 6, GO presents the Raman peak of the *G* band around 1595 cm^−1^ and the *D* band around 1359 cm^−1^, which correspond to the in-plane vibration of *sp* ^2^ carbon atoms and defects in the GO sample, respectively [28]. It is worthwhile to note that the Raman shifts of the *D* and *G*-bands from 1350 cm^−1^ and 1602 cm^−1^ for GO to lower values of 1349 and 1589 cm^−1^ for RGO and RGO/LDH samples confirm the successful in situ reduction of GO [29]. Moreover, the ID/IG value increased gradually from 0.96 for GO, to 1.51 for RGO, and to 1.65 for RGO/LDH confirms the formation of smaller *sp* ^2^ graphitic domains on reduction of GO. The higher ID/IG value for RGO/LDH was ascribed to the higher reduction degree of the GO due to the presence of metal ions [30]. Also, presence of the sufficient defect sites on the surface of graphene can promote nucleation of Zn–Al LDH onto graphene. Moreover, the 2D band at 2677 cm^−1^ was more clearly observed in the RGO/LDH hybrids which confirm the few layer graphene, resulted due to interaction between LDH and graphene, hindering the self-restacking of the graphene sheets.

**Fig. 7:**
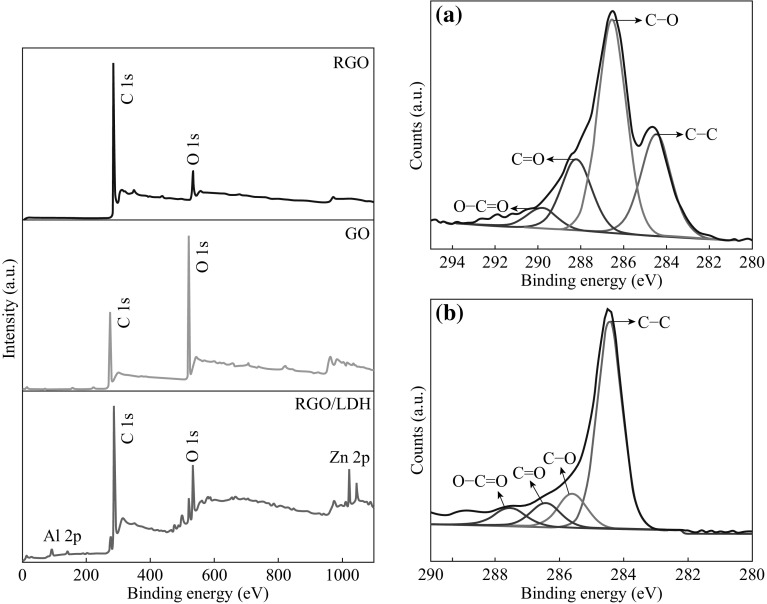
XPS wide scan spectrum of GO, RGO, and RGO/Zn–Al LDH composites (*left*) and C 1*s* XPS spectra of **a** GO, **b** RGO–Zn–Al LDH composites (*right*)

The FT-IR spectra of GO, RGO/Zn–Al LDH nanohybrids, and pristine LDHs are shown in Fig. 7. The strong and broad peak seen in Fig. 7a at 3360 cm^−1^ for all of the samples indicates the presence of surface O–H stretching vibrations of the C–OH groups and water. The other peaks corresponded to oxygen functional groups, such as, carboxyl C=O stretching of COOH groups (1725 cm^−1^), aromatic stretching C=C (1615 cm^−1^), epoxy C–O group stretching (1218 cm^−1^), and alkoxy C–OH group stretching vibrations (1043 cm^−1^) [31]. In comparison with GO (Fig. 7b), the most of the peak intensities corresponding to carbonyl, epoxide, and ether groups disappeared or weakened in the FT-IR spectrum of RGO/Zn–Al LDH, signifying the reduction of oxygen in the as-prepared hybrid nanostructure. Moreover, the bands in lower frequency region below 750 cm^−1^ are accounted for lattice vibration modes for M–O and M–O–M (M–Zn, Al), which also can be seen in FT-IR spectra of pristine Zn–Al LDH. Hence, FT-IR studies confirm the in situ reduction of GO to RGO with simultaneous growth of Zn–Al LDH under microwave irradiation.

### TGA Result

**Fig. 8:**
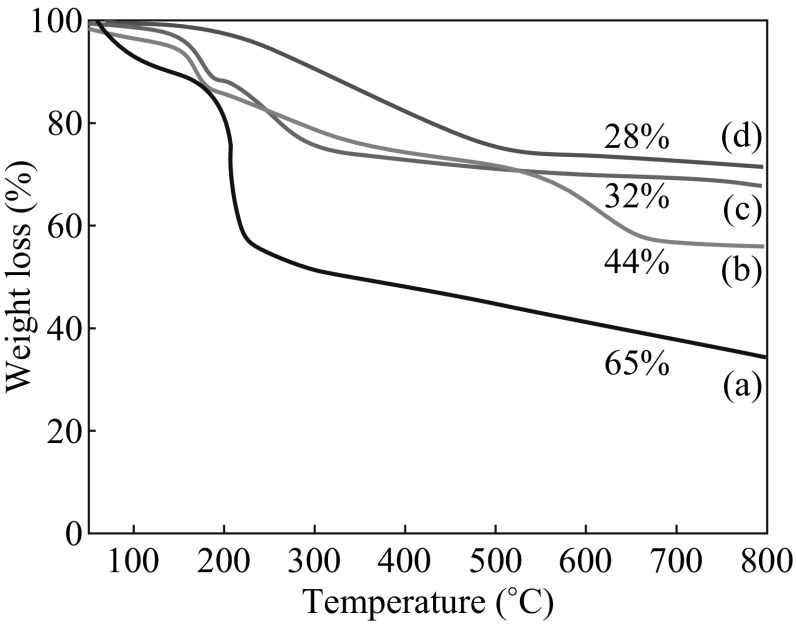
TGA results of* a* GO,* b* Zn–Al LDH,* c* RGO/Zn–Al LDH, and* d* nanohybrids RGO

The TGA behaviors of the graphene/LDH nanohybrids are displayed in Fig. 8a. The pristine GO exhibits single step TGA with significant weight loss at around 210 °C presumably due to the pyrolysis of the labile oxygen-containing functional groups of the carbon skeleton of GO [29]. After reduction, the removal of the thermally labile oxygen functional groups by in situ ammonia reduction results in much increased thermal stability for the RGO (Fig. 8d). However, a minor but gradual mass loss below 600 °C, which can be attributed to the loss of adsorbed water, residual and oxygenated functionalities, was observed. The Zn–Al LDH shows typical thermal decomposition pattern and mainly attributed to water loss (both free and physisorbed) followed by dehydroxylation (Fig. 8b**)** [32]. In the TGA pattern of the RGO/LDH nanohybrids (Fig. 8c), three major weight loss stages were observed. The first weight loss, at approximately 144 °C, is attributed to the removal of loosely bound water molecules from the LDHs interlayer. The second weight loss, in the temperature range 200–400 °C, is due to the removal oxygen functionalities. The third and final weight loss, observed in the temperature above 400 °C, is primarily due to dehydroxylation and decarbonation of the LDH sheets. In presence of urea, the cumulative weight loss for GO is significantly decreased (from 65 to 28 wt%), confirming the in situ reduction of GO to RGO. Interestingly, these TGA results confirm that the urea that was employed as pH-regulator for LDHs synthesis can simultaneously reduce GO to obtain hierarchical graphene/LDH nanohybrids. Moreover, the resulting nanohybrids show typical features of both pristine LDHs and RGO. In other terms, the TGA analysis provides direct evidence of formation of graphene/LDH nanohybrids and efficient reduction of GO in presence of urea under microwave.

### Electrochemical Behaviors

**Fig. 9:**
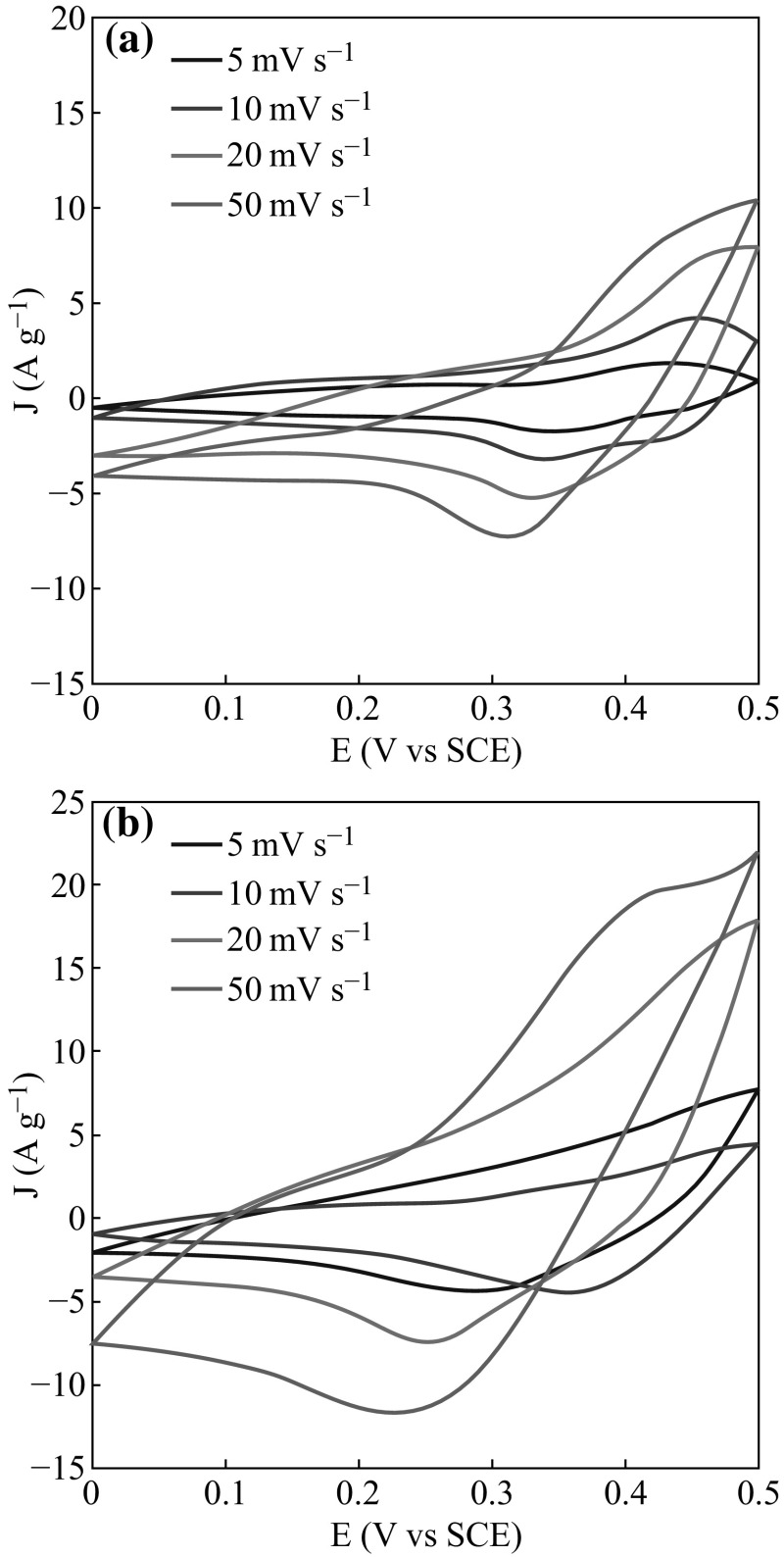
Cyclic voltammetry curves of **a** the pure RGO and **b** RGO/Zn–Al LDH nanohybrids at different scan rates in 1 M KOH solution

To evaluate the electrochemical behaviors of the as-synthesized RGO and RGO/Zn–Al LDH nanohybrids for supercapacitor application, a series of CV and galvanostatic charge/discharge measurements were carried out. Figure 9a, b show the CV curves of the RGO and RGO/Zn–Al LDH nanohybrids at scan rates of 5, 10, 20, and 50 mV s^−1^ in 1 M KOH solution. A couple of anodic/cathodic peaks were observed for CV curves of each sample at different scan rates, including the RGO (Fig. 9a) and RGO/Zn–Al LDHs nanohybrids (Fig. 9b). The results indicate that the presence of LDH significantly alters pseudocapacitance of the graphene in nanohybrids. It is noteworthy that the RGO/Zn–Al LDH nanohybrids show a smaller potential difference (Δ*E*_a,c_) between the anodic and cathodic peaks than RGO under the same scan rate, demonstrating a better electrochemical reversibility of the RGO/Zn–Al LDH hybrids.

**Fig. 10:**
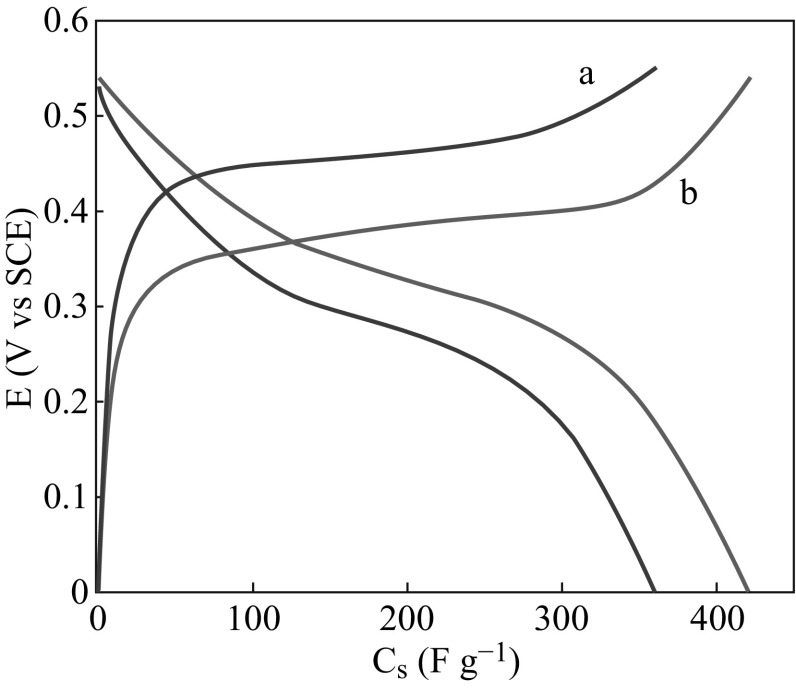
Charge–discharge curves of* a* RGO and* b* RGO/Zn–Al LDH nanohybrids at various discharge current densities

Moreover, the area surrounded by CV curves of the nanohybrids is obviously greater than that of RGO, implying a higher electrochemical capacitance. The charge/discharge curves of RGO and RGO/Zn–Al LDHs at current density of 2 A g^−1^ are presented in Fig. 10. It can be seen that specific capacitance of RGO electrodes is 361.5 F g^−1^ which significantly improved upon hybridization with LDHs. The RGO/Zn–Al LDHs exhibit prominent discharge specific capacitance 428 F g^−1^, which is almost 20 % higher than RGO. The improved capacitive behavior is mainly attributed to the high electronic conductivity of the RGO/Zn–Al LDH nanohybrids, which comes from two sources of intrinsic high conductivity of the graphene sheets and interaction between the RGO and the LDHs in the nanohybrids. The anchored LDH particles play an active role preventing the aggregation of the graphene sheets, which connect with each other to further establish a conductive network for facile electron transport. The high specific surface area of the RGO/Zn–Al LDH hybrids favors the electrolyte percolation to achieve high accessibility of the active LDHs materials, which also contributes to improved specific capacitance.

## Conclusion

Microwave-assisted method for hybridizing graphene with LDHs is presented. This efficient and rapid process involves the in situ reduction of GO to graphene with simultaneous growth of 2D Zn-containing LDHs using urea hydrolysis under microwave conditions. The resulting hybrids show improved electrochemical-specific capacitance, which attained maximum of 428 F g^−1^, almost 20 % higher than that of pristine graphene. Moreover, the RGO/Zn–Al LDH hybrids demonstrated good cycling stability. Hence, LDH nanosheets in conjunction with thermo-electroconductive graphene endow the resulting hybrid nanocomposites with new multifunctional properties. The effectiveness of the method described above for fabricating graphene/LDH nanohybrids could be of high importance for the preparation of other graphene-based hybrid nanocomposite electrode materials. Similarly, the use of microwave in fabrication of graphene/LDH nanohybrids provides a novel method for the development of new multifunctional nanocomposites on the basis of the existing nanomaterials.
